# Multidisciplinary management and outcomes of abdominal vascular graft and endograft infections: a retrospective cohort study

**DOI:** 10.1007/s15010-026-02885-w

**Published:** 2026-07-15

**Authors:** Anna Juul Christensen, Stephanie Bjerrum, Karl Sörelius, Inger Hee Mabuza Mathiesen, Katja Vogt, Claus Moser, Jannik Helweg-Larsen, Benjamin Vikjaer Sandholt, Daniel Faurholt-Jepsen, Christian Kraef

**Affiliations:** 1 Department of Infectious Diseases, Copenhagen University Hospital - Rigshospitalet, Copenhagen, Denmark; 2 Department of Respiratory Medicine and Infectious Diseases, Copenhagen University Hospital - Nordsjaellands Hospital, Hilleroed, Denmark; 3 Department of Clinical Medicine, University of Copenhagen, Copenhagen, Denmark; 4 Department of Vascular Surgery, Copenhagen University Hospital - Rigshospitalet, Copenhagen, Denmark; 5 Department of Clinical Microbiology, Copenhagen University Hospital - Rigshospitalet, Copenhagen, Denmark; 6 Department of Immunology and Microbiology, University of Copenhagen, Copenhagen, Denmark

**Keywords:** Antimicrobial therapy, Aortic vascular graft and endograft infection, Biofilm infections, Microbiology, Implant-associated infections

## Abstract

**Purpose:**

To describe the multidisciplinary management and outcomes of patients with vascular graft and endograft infections of the abdominal aorta (aVGEI) in Eastern Denmark.

**Methods:**

Retrospective cohort study of aVGEI patients between 1st July 2022 and 30th June 2025. Diagnosis and management were based on a multidisciplinary team (MDT) conference. The primary outcome was 90-day mortality. Secondary outcomes included one-year mortality, cure, remission, treatment failure, and re-hospitalization.

**Results:**

In 35 aVGEI patients, the median age was 73 years (IQR 69.5–79 years), and the median Charlson Comorbidity Index score was 5 (IQR 3–6). The Management of Aortic Graft Infection Collaboration (MAGIC) criteria were fulfilled in 27 cases (77%, ‘MAGIC positive aVGEI’). The remaining eight cases (23%) were based on conference consensus, often supported by positive PET-CT findings (*n* = 4/8). The most frequent monomicrobial infections were *Staphylococcus aureus* (*n* = 5/35, 14.3%) and coagulase-negative staphylococci (*n* = 4/35, 11.4%). Polymicrobial infections occurred in nine patients (25.7%). Five aVGEI patients were treated surgically (14.3%). The 90-day mortality was 8.6% (*n* = 3/35, 95%CI 1.8–3.1%); 4.2% among ‘MAGIC positive aVGEI’ patients (*n* = 1/24), 12.5% among those diagnosed on conference consensus (*n* = 1/8), and 33.3% among patients with an aortoenteric fistula (AEF) (*n* = 1/3). Among aVGEI patients with complete follow-up, cure was achieved in 31.8% (*n* = 7/22, 95%CI 13.9–4.9%), but none of the three patients with AEF.

**Conclusion:**

We report low short-term mortality and a cure rate of over 30% within a cohort of predominantly conservatively managed patients discussed at an MDT conference. These outcomes may reflect the benefits of individualized treatment decisions based on careful patient evaluation during MDT meetings as well as survivor bias.

**Supplementary Information:**

The online version contains supplementary material available at https://doi.org/10.1007/s15010-026-02885-w.

## Introduction

Vascular graft and endograft infections (VGEI) are rare clinical complications to vascular surgery, but pose significant diagnostic and therapeutic challenges due to their complex nature and high rates of illness [1]. Abdominal VGEI (aVGEI) are a distinct category of VGEI with particularly high mortality and morbidity [2].

Establishing a diagnosis of VGEI relies on a comprehensive evaluation of clinical, laboratory and radiological examinations, sometimes coupled with intraoperative evidence. In 2016, the Management of Aortic Graft Infection Collaboration (MAGIC) defined diagnostic criteria based on a multidisciplinary expert review to standardize diagnosis of VGEI [3]. Treatment of VGEI frequently requires a combination of prolonged antibiotic therapy and/or complex surgery, probably due to biofilm formation on the alloplastic material [4], and definite cure often depends on graft explantation with thorough debridement - a mandatory approach in cases complicated by a fistula [1, 5]. There is no standardised regimen for empirical antibiotic treatment, as the choice of therapy depends on the local epidemiology of microorganisms and patterns of antibiotic resistance. This necessitates an individualized strategy in planning the treatment for the patient. The infrequency and complex management of VGEI illustrates the usefulness of a standardized multidisciplinary team (MDT) approach for its diagnosis and management [6] similar to the well-established concept of endocarditis teams [5, 7, 8].

The absence of randomized controlled trials and limited number of larger cohort and population-based studies leads to scarce availability of reliable data on the characteristics, incidence, risk factors, causative microbiological agents, management strategies as well as the treatment outcomes for VGEI [9]. Such data are crucial to standardize and improve the management of VGEI. To date, no cohorts or case series of VGEI have been reported from Denmark.

The aim of this study was to describe the MDT management, treatment strategies and outcomes of patients with aVGEI in Eastern Denmark.

## Methods

### Study design and participants

We conducted a retrospective cohort study of patients with aVGEI diagnosed between 1st July 2022 and 30th June 2025 at an MDT conference. The Department of Vascular Surgery at Rigshospitalet, Copenhagen University Hospital is the only department for vascular surgery in the Capital Region of Denmark. Of note, Denmark offers universal health care to all citizens. All patients in the Capital Region suspected of a VGEI are eligible to be discussed at the MDT conference and all patients evaluated at the VGEI MDT conference were assessed for inclusion in this study. The MDT conference at Rigshospitalet, Copenhagen covers all of the Eastern Region in Denmark, similar conferences exist at tertiary centres in both the Southern and Central Regions of Denmark which are not a part of this study. In the event of treatment failure, only the first instance of infection was included in the analysis. The bi-weekly conference includes infectious diseases specialists, clinical microbiologists, and vascular surgeons.

The primary outcome was 90-day mortality from diagnosis among aVGEI patients. Secondary outcomes were cure of disease, remission of disease, and treatment failure at one-year follow-up as well as one-year mortality. Furthermore, the study evaluated re-hospitalisation of patients with aVGEI at one-year follow-up.

### Definitions

Patients were categorized by diagnosis as ‘MAGIC positive aVGEI’ if they fulfilled MAGIC criteria, or as ‘clinical aVGEI’ if defined as aVGEI by MDT consensus decision and not fulfilling MAGIC criteria. All included aVGEI were initially diagnosed based on a clinical evaluation and consensus at the MDT conference and MAGIC criteria were applied retrospectively. A patient was categorised as ‘MAGIC positive aVGEI’ when a single major criterion was present at the same time as any other criterion (major or minor) from another category as described by Lyons et al. [3, 10]. If the patient only fulfilled minor criteria but was diagnosed by the MDT conference, the patient was categorised as ‘clinical aVGEI’. Furthermore, each patient was categorized as being either aVGEI or aVGEI with an aorto-enteric fistula (AEF).

To evaluate the time-point of diagnosis, patients were categorised into early or late aVGEI. In this study, early infection was defined as diagnosis ≤ 120 days from index surgery, based on MAGIC criteria [3]. When the MAGIC criteria were not fulfilled, first suspicion of infection described in the records was used as the timepoint of diagnosis. Late infection was defined as > 120 days from index surgery to date of diagnosis [3, 10].

The Charlson Comorbidity Index (CCI) was used to describe patients’ comorbidities [11]. Only microorganisms deemed clinically relevant by the MDT conference were included in the analysis, i.e., microorganisms that were interpreted as contamination were excluded. Polymicrobial infections were defined as ≥ 2 causative microbiological agents in the same patient whereas monomicrobial infections were defined as one causative microbiological agent.

The index surgery was defined as the surgery in which the graft which was later infected was primarily inserted.

Empirical antibiotic treatment was defined as antimicrobial treatment initiated before the causative microorganism(s) were identified. Targeted treatment was defined as either a continuation or switch of antibiotic treatment after the identification of the causative microbiological agents. The treatment strategy in relation to antimicrobial therapy was categorised as either suppressive or curative based on electronic patient records. Curative treatment was defined as antimicrobial treatment with the potential to eradicate the infection and was used when the goal was to eliminate the infection. Suppressive treatment was defined as antimicrobial treatment intended to suppress the infection, without the potential to eradicate the infection due to negative culture results and/or biofilm formation not possible to treat. Suppressive treatment was used in patients deemed unfit for surgery and in need of long-term, usually lifelong, antibiotic treatment.

Surgical treatment was categorised as either surgical drainage, endovascular repair, partial or total graft removal with extra-anatomical bypass (EAB) or in situ reconstruction. Conservative treatment was defined as exclusive antibiotic treatment with or without therapeutic percutaneous drainage.

Patients were followed for a minimum of 12 months after aVGEI diagnosis with outcomes categorized into either cure of disease, remission of disease, treatment failure, or death consistent with definitions in a recent Delphi consensus paper [12]. Cure of aVGEI was defined as being achieved one year after cessation of antimicrobial therapy, without any signs of relapse of infection on follow-up including clinical evaluation, laboratory tests, or imaging. Remission of aVGEI was defined as a patient without symptoms and without deterioration on clinical examination, laboratory tests or imaging on continued suppressive antibiotic treatment for more than one year [5]. Treatment failure of aVGEI was defined as the failure to achieve cure or remission one year after aVGEI diagnosis.

Outcome analyses were separated into ‘MAGIC positive aVGEI’ and ‘clinical aVGEI’, respectively. MAGIC positive aVGEI with an aortoenteric fistula (AEF) were also analysed separately as they represent a pathophysiologically, clinically, and prognostically distinct entity.

For descriptive statistics, only patients with full follow-up at 12 months were included in the outcome analyses for the one-year mortality proportion as well as for cure, remission and treatment failure as defined above. For Kaplan-Meier Plots, all aVGEI patients were included, accounting for censoring.

### Data extraction and statistical analysis

Data was extracted from patients’ electronic health records and entered into a secure predefined Research Electronic Data Capture (REDCap) database [13] containing information on patient characteristics, risk factors, the infection including biochemistry, treatment, and outcome. Continuous variables are presented as median with interquartile range according to data distribution. Missing values were excluded from the analysis.

The study was reported in accordance with the STROBE (Strengthening the Reporting of Observational Studies in Epidemiology) checklist for cohort studies (Supplementary Table 1). Data management and analysis was performed using R statistical software version 2025.05.1 + 513 for MacOS [14].

### Ethical considerations

The project was approved by Copenhagen University Hospital with the approval number p-2024-17779 as a quality improvement project.

## Results

**Fig. 1:**
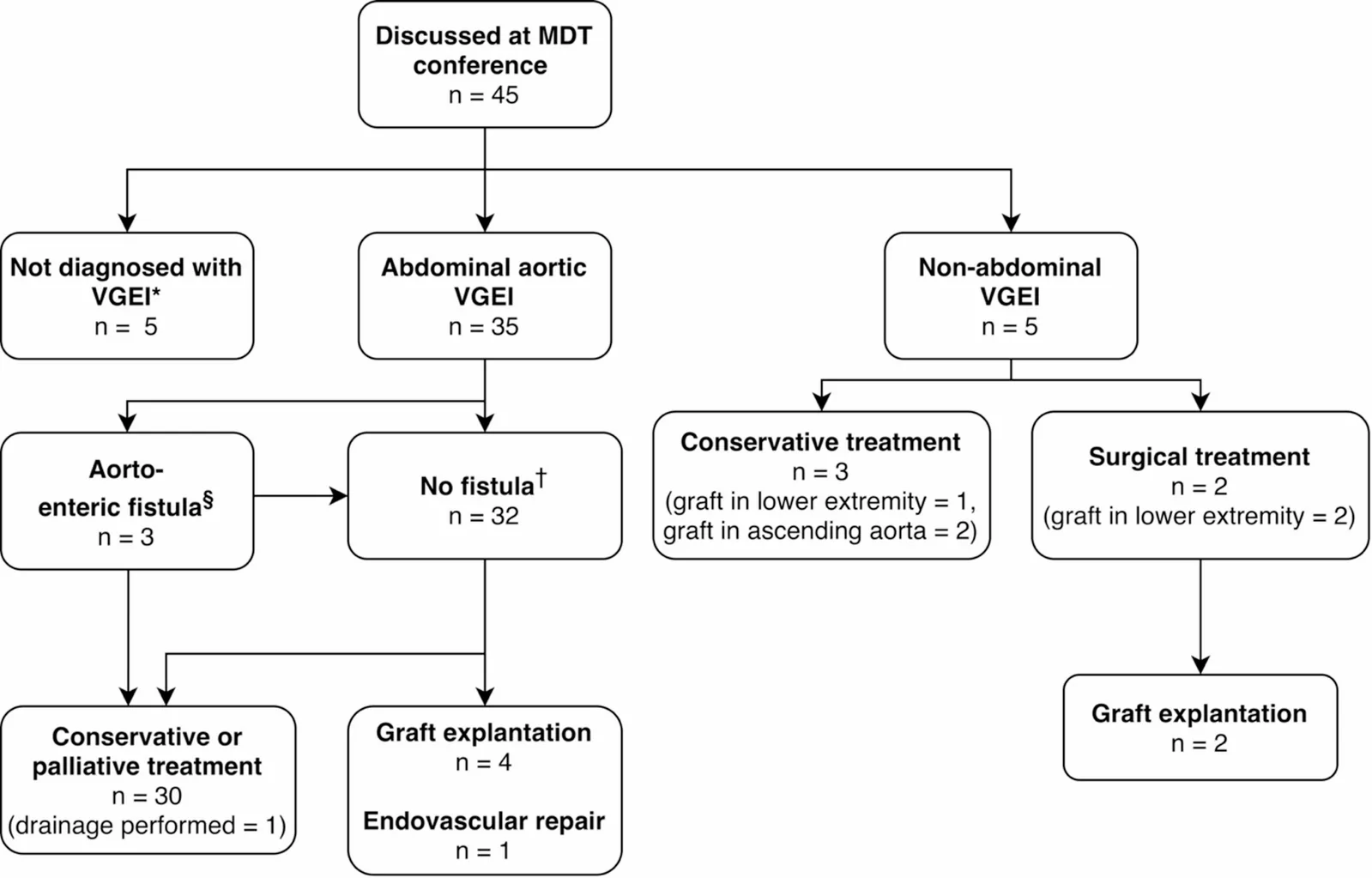
Flowchart of the treatment strategy for VGEI patients discussed at the multidisciplinary team (MDT) conference. *The patients not diagnosed with VGEI were based on a multidisciplinary team conference decision and the diagnosis was dismissed either due to no microbiological agent being found, suspicion of aseptic aortitis or spondylodiscitis, and/or negative PET-CT result. ^§^All patients with aorto-enteric fistula underwent conservative treatment strategy due to not being fit for surgery. ^†^Four patients had conservative treatment even though they were deemed fit for surgery. Three of these cases were due to sufficient effect of antimicrobial therapy, one case declined surgery

A total of 45 patients suspected of having a VGEI were discussed at the MDT conference (Fig. 1). Of those, 40 patients were diagnosed with VGEI: 35 aVGEI and five non-abdominal VGEI patients (non-aVGEI were not included in this paper). Three aVGEI patients had aorto-enteric fistulation (*n* = 3/35, 8.6%).

### Patient characteristics

The median age of all aVGEI patients was 73 years (IQR 69.5–79 years), and most participants were male (*n* = 23/35, 65.7%) (Table 1). Frequent comorbidities were chronic pulmonary disease (*n* = 13/35, 37.1%), peripheral vascular disease (*n* = 12/35, 34.3%), and diabetes mellitus (*n* = 9/35, 25.7%). The median overall CCI was 5 (IQR 3–6); 6 (IQR 5–7) among ‘clinical aVGEI’, 4.5 (IQR 4–6) among ‘MAGIC positive aVGEI’, and 7 (IQR 4.5–7.5) among aVGEI with an AEF. Eight patients (*n* = 8/35, 22.6%) had more than one indication for the index surgery. The most common indication for the index surgery was aneurism (*n* = 19/35, 54.3%), followed by occlusive disease (*n* = 9/35, 25.7%), and infective native aortic aneurism (*n* = 7/35, 20%). Biomarkers at time of aVGEI diagnosis were generally elevated with a median C-reactive protein (CRP) of 98 mg/L (IQR 64–167.5 mg/L) and a total leucocyte count of 10.6 10^9^/L (median, IQR 8.25–12.80 10^9^/L). Procalcitonin, however, was low with a median value of 0.28 ng/mL (IQR 0.12–0.53 ng/mL), although missing data was substantial (31%). Twelve patients presented with fever (*n* = 12/35, 34.3%), and 12 patients presented with localized clinical features such as swelling, redness or pain (*n* = 12/35, 34.3%). Patients were hospitalized for a median of 25 days (IQR 13.5–35.5 days), most often without being admitted to the Intensive Care Unit (ICU) (*n* = 26/35, 74.3%). The aVGEIs were categorized as early infection in 42.9% (*n* = 15/35) and late infection in 57.1% of patients (*n* = 20/35).

**Table 1:** Characteristics of all patients with aortic vascular graft and endograft infections (aVGEI)

	Total aVGEI *n* = 35 *n* (%)	MAGIC positive aVGEI without AEF *n* = 24 *n* (%)	Clinical aVGEI *n* = 8 *n* (%)	aVGEI with AEF *n* = 3 *n* (%)
Age (in years), median (IQR)	73 (69.5–79)	71.5 (68.75–77.50)	77.5 (72.75–83.25)	70 (59–72)
Sex, male	23 (65.7)	14 (58.3)	7 (87.5)	2 (66.7)
Comorbidities				
Previous myocardial infarction	7 (20.0)	3 (12.5)	3 (37.5)	1 (33.3)
Congestive heart failure^a^	7 (20.0)	4 (16.7)	2 (25.0)	1 (33.3)
Peripheral vascular disease	12 (34.3)	8 (33.3)	2 (25.0)	2 (66.7)
Cerebrovascular disease	3 (8.6)	2 (8.3)	1 (12.5)	0 (0)
Chronic pulmonary disease^b^	13 (37.1)	7 (29.2)	4 (50.0)	2 (66.7)
Liver disease	0 (0)	0 (0)	0 (0)	0 (0)
Diabetes mellitus	9 (25.7)	7 (29.2)	2 (25.0)	0 (0)
HbA1c (mmol/mol), median (IQR)	47 (39–54)	50 (44.5–59.5)	36.5 (36.25–36.75)	–
Renal disease^c^	7 (20)	4 (16.7)	2 (25.0)	1 (33.3)
eGFR (mL/min/1.73 m^2^), median (IQR)	36 (16.50–41.50)	28.5 (13–43.50)	38 (37–39)	19 (19–19)
Any malignancy (solid or haematological)	4 (11.4)	2 (8.3)	1 (12.5)	1 (33.3)
Immune disorder^d^	5 (14.3)	2 (8.3)	2 (25.0)	1 (33.3)
CCI, median (IQR)	5 (3–6)	4.5 (4–6)	6 (5–7)	7 (4.5–7.5)
Recent smoking, yes^e^	16 (45.7)	14 (58.3)	0 (0)	2 (66.7)
Ever smoking, yes	32 (91.4)	22 (91.7)	8 (100)	2 (66.7)
Number of pack years, median (IQR)^f^	40 (21–54.50)	41.5 (28.75–52.75)	14 (12–38)	57 (53.5–60.5)
Type of index surgery^g^				
Endovascular repair	17 (50)	12 (52.2)	5 (62.5)	0 (0)
Open surgery	17 (50)	11 (47.8)	3 (37.5)	3 (100)
Groin incision at index surgery, yes^h^	22 (64.7)	15 (65.2)	6 (75.0)	1 (33.3)
Post index surgery wound complications^i^	13 (38.2)	9 (39.1)	3 (37.5)	1 (33.3)
Superficial infection	7 (53.8)	5 (55.6)	2 (66.7)	0 (0)
Deep infection	2 (15.4)	0 (0)	2 (66.7)	0 (0)
Hematoma	5 (38.5)	3 (33.3)	2 (66.7)	0 (0)
Other^j^	4 (30.8)	2 (22.2)	1 (33.3)	1 (100)
Duration of hospitalization (days), median (IQR)	25 (13.50–35.50)	25 (13.75–34)	33.5 (23.75–39.75)	13 (11–40.5)
Biomarkers, median (IQR)				
CRP (mg/L)	98 (64–167.5)	92.5 (34.25–157.75)	114 (76–156.5)	117 (100–196.5)
Leucocytes (10^9^/L)	10.60 (8.25–12.80)	10.40 (8.80–12.65)	10.80 (7.45–12.12)	11.40 (8.85–12.25)
Procalcitonin (ng/mL)^k^	0.28 (0.12–0.53)	0.29 (0.13–0.50)	0.11 (0.05–5.65)	0.41 (0.33–0.53)
Time-point of diagnosis				
Early aVGEI (≤ 120 days since surgery)	15 (42.9)	12 (50.0)	2 (25.0)	1 (33.3)
Late aVGEI (> 120 days since surgery)	20 (57.1)	12 (50.0)	6 (75.0)	2 (66.7)

*AEF* Aorto-enteric fistula, *IQR* Interquartile range, *aVGEI* Aortic vascular graft and endograft infection, *HbA1c* Haemoglobin A1c, *eGFR* Estimated glomerular filtration rate, *CCI* Charlson Comorbidity Index, *CRP* C-reactive protein

^a^Including heart failure with preserved, reduced, and mildly reduced ejection fraction

^b^Including chronic obstructive pulmonary disease (COPD), asthma, obstructive sleep apnea, and pulmonary hypertension

^c^Defined as an estimated glomerular filtration rate (eGFR) < 45

^d^Including regular corticosteroid administration or immunosuppressive medication, including AIDS (*n* = 0)

^e^Defined as regular smoking within five years of aVGEI diagnosis

^f^Missing values = 9 (6 from MAGIC positive aVGEI group and 3 from clinical aVGEI group, respectively)

^g^Missing values = 1 (from MAGIC positive aVGEI group)

^h^Missing values = 1 (from MAGIC positive aVGEI group)

^i^Missing values = 1 (from MAGIC positive aVGEI group)

^j^Including necrosis and lymphoma/seroma

^k^Missing values = 11 (9 from MAGIC positive aVGEI group and 2 from clinical aVGEI group, respectively)

### Outcomes

Among all aVGEI patients the total 90-day mortality was 8.6% (*n* = 3/35, 95%CI 1.8–23.1%) (Table 2). There was one death among ‘MAGIC positive aVGEI’ patients (*n* = 1/24, 4.2%), one among aVGEI with an AEF (*n* = 1/3, 33%), and one among ‘clinical aVGEI’ (*n* = 1/8, 12.5%). The overall median time to death was 170 days (IQR 33.5–30.5 days).

Of 22 aVGEI patients with complete follow-up, six had died (27.3%, 95%CI 10.7–50.2%), seven were cured (31.8%, 95%CI 13.9–54.9%), eight were in remission (36.7%, 95%CI 17.2–59.3%), and one was considered treatment failure (4.5%, 95%CI 0.1–22.8%). Thirteen patients (*n* = 13/35, 37%) lack complete follow-up due to recent diagnosis and were not included in the one-year outcome analyses. Outcomes for ‘MAGIC positive aVGEI’ and ‘clinical aVGEI’ patients as well as patients with an AEF are provided in Table 2.

Supplementary Figs. 1 and 2 show Kaplan-Meier plots of overall one-year survival among all aVGEI patients and ‘MAGIC positive aVGEI’ patients, respectively.

Over half of aVGEI patients had been re-hospitalized at one-year follow-up (*n* = 13/22, 59.1%) with a median time from discharge to re-hospitalization of 75 days (IQR 35–95 days).

**Table 2:** Outcomes in patients with abdominal vascular graft and endograft infections (aVGEI)

	Total *n* = 35 *n* (%)	MAGIC positive aVGEI *n* = 24 *n* (%)	Clinical aVGEI *n* = 8 *n* (%)	Aorto-enteric fistula *n* = 3 *n* (%)
Primary outcome 90-day mortality	3 (8.6)	1 (4.2)	1 (12.5)	1(33.3)
Secondary outcomes^a^				
Cure (*n* = 22)	7 (31.8)	5 (35.7)	2 (40.0)	0 (0)
Remission (*n* = 22)	8 (36.7)	6 (42.9)	1 (20.0)	1 (33.3)
Treatment failure (*n* = 22)	1 (4.5)	0 (0)	1 (20.0)	0 (0)
30-day mortality	2 (5.7)	1 (4.2)	0 (0)	1(33.3)
1-year mortality (*n* = 22)	6 (27.3)	3 (21.4)	1 (20.0)	2 (66.7)
1-year re-hospitalization (*n* = 22)	13 (59.1)	8 (57.1)	4 (80.0)	1 (33.3)

^a^Thirteen patients lack full follow-up due to recent diagnosis. The secondary outcome analysis was performed on the remaining 22 patients having full follow-up. Among MAGIC positive aVGEI, 14 patients have full follow-up and among clinical aVGEI, five patients have full follow-up. All with aorto-enteric fistulation have full follow-up

### Diagnosis

Table 3 provides an overview of the included patients by MAGIC criteria fulfilled (‘MAGIC positive aVGEI’ vs ‘clinical aVGEI’). For ‘MAGIC positive aVGEI’ patients, the most frequent major criterion fulfilled was graft insertion in an infected site (*n* = 9/13, 75.0%) among early aVGEI patients, and peri-graft fluid on CT-scan ≥ 3 months after insertion (*n* = 5/14, 35.7%) among patients with late aVGEI. Regarding minor criteria, abnormally elevated inflammatory markers was the most common criterion in both early (*n* = 13/13, 100%) and late (*n* = 13/14, 92.9%) ‘MAGIC positive aVGEI’ patients. Among patients with ‘clinical aVGEI’, “other radiology” was the most frequent minor MAGIC criterion (*n* = 7/8, 87.5%). This category consisted mainly of suspicious metabolic activity on F^18^ Flourodeoxyglucose (FDG) Positron Emission Tomography-Computed Tomography (PET-CT) (*n* = 4/7, 57.1%) as well as suspicious tissue inflammation (*n* = 3/7, 42.9%), aneurysm formation (*n* = 3/7, 42.9%), and discitis/osteomyelitis (*n* = 3/7, 42.9%). Regarding patients with positive finding on FDG-PET-CT, three out of four had a late aVGEI whereas one had an early aVGEI.

**Table 3:** Presence and distribution of MAGIC criteria among all patients with abdominal vascular graft and endograft infections (aVGEI) including cases with an aorto-enteric fistula

MAGIC criteria fulfilled	MAGIC positive aVGEI (*n* = 27)		Clinical aVGEI
	Early infection *n* = 13 (48.1%) *n* (%)	Late infection *n* = 14 (51.9%) *n* (%)	(*n* = 8) *n* (%)
Clinical/surgical			
*Major*			
Pus	0 (0)	3 (21.4)	0 (0)
Open wound	2 (15.4)	2 (14.3)	0 (0)
Fistula	1 (7.7)	2 (14.3)	0 (0)
Infected site	9 (75.0)^a^	1 (7.1)	0 (0)
*Minor*			
Localized clinical feature	3 (23.1)	7 (50.0)	2 (25.0)
Fever	2 (15.4)	6 (42.9)	4 (50.0)
Radiology			
*Major*			
Peri-graft fluid	1 (7.7)	5 (35.7)	0 (0)
Peri-graft gas	0 (0)	4 (28.6)	0 (0)
Increase in peri-graft gas	0 (0)	0 (0)	0 (0)
*Minor*			
Other	4 (30.8)^b^	12 (85.7)^c^	7 (87.5)^d^
Laboratory			
*Major*			
Organisms from graft	0 (0)	3 (21.4)	0 (0)
Organisms from specimen	5 (38.5)	2 (14.3)	0 (0)
Organisms from aspirate	1 (7.7)	3 (21.4)	0 (0)
*Minor*			
Positive blood culture	3 (23.1)	6 (42.9)	3 (37.5)
Elevated bio markers	13 (100)	13 (92.9)	7 (87.5)
Major criteria fulfilled, median (IQR)	1 (1–2)	1 (1–2)	0 (0–0)
Minor criteria fulfilled, median (IQR)	2 (1–2)	3 (3–3)	3 (2–4)

^a^Missing value = 1

^b^One of four (25%) was positive on PET-CT

^c^Five of 12 (41.7%) were positive on PET-CT

^d^Four of seven (57.1%) were positive on PET-CT

### Treatment

All patients received antimicrobial therapy. Empirical treatment was initiated in 22 aVGEI patients (*n* = 22/35, 62.9%), usually followed by switch to targeted treatment (*n* = 15/22, 68.2%). A combination of meropenem 2 g TID and vancomycin 1 g BID was used for empirical therapy in 40.9% (*n* = 9/22) of cases. Excluding patients who died in-hospital (*n* = 3) the median duration of intravenous (IV) administration was 30 days (IQR 18.25–44 days). Among ‘MAGIC positive aVGEI’ patients without AEF, the median duration of IV treatment was 29 days (IQR 15.5–46 days) compared with ‘clinical aVGEI’ who received IV treatment for a median period of 33.5 days (IQR 22.25–44 days).

Almost all (*n* = 30/35, 85.7%) were switched to oral antibiotics after the initial period on IV treatment. The most used oral antibiotic regimens were the combinations of rifampicin 600 mg BID and amoxicillin 1 g QID (*n* = 3/30, 10%), moxifloxacin 400 mg once daily and linezolid 600 mg BID (*n* = 3/30, 10%), and moxifloxacin 400 mg once daily as monotherapy (*n* = 3/30, 10%). Reasons for not receiving oral treatment included in-hospital mortality (*n* = 3/5, 60%), or sufficient clinical and biochemical response to treatment during the IV treatment period leading to discontinuation of antibiotic therapy (*n* = 2/5, 40%). The aim of the final antimicrobial therapy was curative in 22 cases (*n* = 22/35, 62.9%) and suppressive in 13 cases (*n* = 13/35, 37.1%). Ten patients were prescribed lifelong suppressive antibiotics (*n* = 10/35, 28.6%). One patient with an AEF was treated conservatively with therapeutic percutaneous drainage in addition to antimicrobial therapy. The remaining two patients with AEF were treated conservatively as surgery could not be performed due to either technical difficulties or comorbidities. For all ‘MAGIC positive aVGEI’ with or without AEF, the aim was curative in 18 cases (*n* = 18/27, 66.7%) compared to ‘clinical aVGEI‘ where the aim was curative in 4 cases (*n* = 4/8, 50%).

**Fig. 2:**
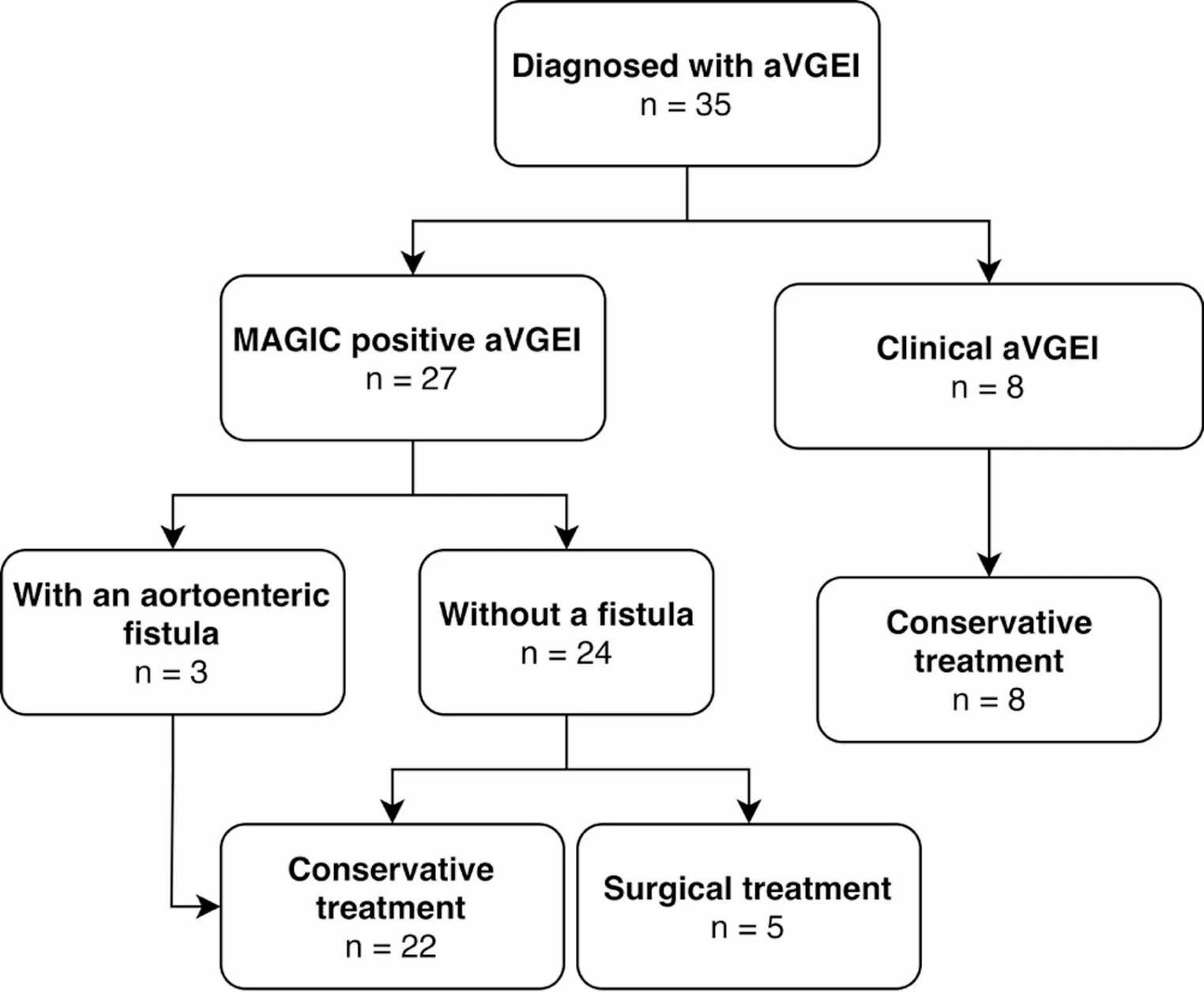
Flowchart of diagnosis based on MDT conference decisions and MAGIC criteria

Five aVGEI patients (*n* = 5/35, 14.3%) underwent surgical treatment in addition to antimicrobial therapy, none of whom had an AEF (Fig. 2). All surgical treated patients were ‘MAGIC positive’. One patient underwent endovascular repair, two patients had partial graft removal with in situ reconstruction, and two patients had total graft removal with in situ reconstruction (Supplementary Table 3). For all patients with graft removal, reconstruction was performed with either bovine pericardium (*n* = 2/4, 50%) or autologous vein (*n* = 2/4, 50%) to prevent re-infection. The median time from index surgery to surgical treatment of aVGEI was 469 days (IQR 461–1167 days), and a median of 9 days from diagnosis to surgery (IQR 3–15 days).

### Microbiology

**Fig. 3:**
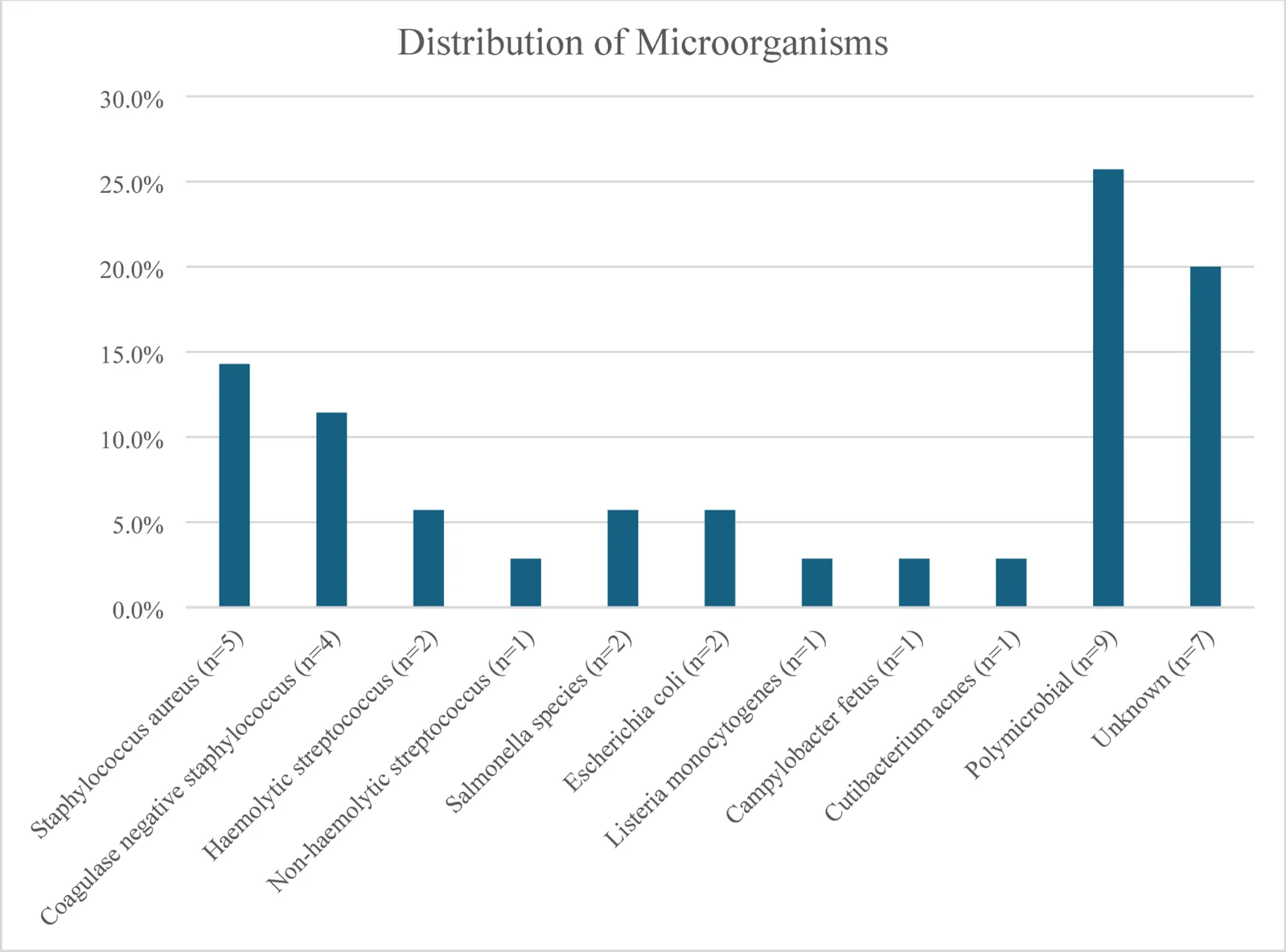
Microorganisms among aVGEI identified by culture or 16 S RNA analysis, deemed as causative by multidisciplinary team conference based on treatment decision. Monomicrobial If possible, this sentence should be moved to the figure description above the figure. infections are illustrated in the below figure as individual microorganisms whereas microorganisms in polymicrobial infections can be seen in Table 4

Infections were monomicrobial in 54.3% (*n* = 19/35), polymicrobial in 25.7% (*n* = 9/35), and of unknown microbiological aetiology in 20% (*n* = 7/35) of cases (Fig. 3). Polymicrobial infections can be seen in Table 4. The most frequently identified causative microorganisms in monomicrobial infections were *Staphylococcus aureus* (*n* = 5/35, 14.3%) and coagulase negative staphylococci (CoNS): two of which were *Staphylococcus epidermidis* (*n* = 2/35, 5.7%), one *Staphylococcus lugdunensis* (*n* = 1/35, 2.9%), and one case of undefined CoNS (*n* = 1/35, 2.9%) (Supplementary Table 2). *Salmonella *species were found in two patients with monomicrobial infection (*n* = 2/35, 5.7%), *Escherichia coli* in two patients (*n* = 2/35, 5.7%), and haemolytic *streptococci* in two patients (*n* = 2/35, 5.7%). Blood culture was the main sample type yielding positive culture results (*n* = 16/35, 45.7%). Other sources included aneurysm tissue (*n* = 6/35, 17.1%), graft material (*n* = 5/35, 14.3%), and wound material (*n* = 8/35, 22.9%)^1^. 16 S-ribosomal ribonucleic acid (rRNA) analysis was done on sterile tissue, pus or aspirate in nine cases (*n* = 9/35, 25.7%). Of all culture negative aVGEI, over half had a polymerase chain reaction (PCR) test performed (*n* = 6/11, 54.5%) with four of these PCR analyses yielding positive results and two remaining negative.

**Table 4:** Combinations of microorganisms in the polymicrobial infections (*n* = 9)

	Microorganisms
I	Non-haemolytic streptococcus, Eschericia coli, Candida tropicalis, and Lactobacillus germentum
II	*Streptococcus mitis* and Anaerococcus species
III	*Staphylococcus aureus*, *Klebsiella oxytoca*, *Acinebacter spp.*, and Haemolytic streptococcus group B
IV	*Staphylococcus epidermidis* and *Staphylococcus capitis*
V	*E. coli* and Parvimonas species
VI	*E. coli*, *Enterococcus faecium* (VLRE), *Klebsiella* spp., *Lactobacillus germentum*, and *Candida Glabrata*
VII	*E. faecalis* and *Corynebacterium jeikeium*
VIII	*S. epidermidis* and *Peptoniphilus harei*
IX	*S. epidermidis* and *Cutibacterium acnes*

*VLRE* Vancomycin and Linezolid resistant enterococcus

## Discussion

This retrospective cohort study from Eastern Denmark included 35 patients with aVGEI, of which 27 were ‘MAGIC positive’. Despite relatively advanced age and high multimorbidity, the observed 90-day mortality was only 8.6% (95%CI 1.8–23.1%), although this estimate derives from a small sample of 35 patients, resulting in wide confidence intervals. One-year mortality increased to 27% among the 22 aVGEI patients with complete follow-up, which is at the lower end of other published cohort studies, varying between 12% and 55% [2, 15, 16]. This is, to the best our knowledge, the first systematic description of characteristics, management and outcomes of a cohort of patients with aVGEI from Denmark.

This Danish cohort was characterized by high age (median 73 years) combined with multimorbidity (CCI median 5) comparable to previously published cohorts from Sweden, France, Switzerland, and the United States (US) [2, 5, 15–18]. Interestingly, about one in four patients in our study was diagnosed based on a clinical evaluation by MDT consensus and did not fulfil MAGIC criteria. A previous validation study found a sensitivity of 99% and a specificity of 61% for MAGIC criteria compared to a multidisciplinary consensus decision [19]. The relatively large proportion of patients not fulfilling MAGIC criteria, may be reflecting the fact that the patients were diagnosed by the MDT, and that the MAGIC criteria were applied retrospectively for the purpose of this study. In the majority of the patients clinically diagnosed with aVGEI by MDT consensus, the eventual diagnosis was supported by PET-CT results, essentially elevating a positive PET-CT finding from a minor to a major criterion in the decision-making process [7, 20]. One patient from our study with positive findings on PET-CT had an early infection and the results should therefore be interpreted with caution. However, the scans were discussed at the MDT conference before deemed clinically significant. A study by Dong et al. compared the result of PET-CT with diagnosis based on MAGIC criteria and found a high concordance between the two diagnostic methods (88%) [21]. Attention should be paid to focality of the FDG uptake, using a five-point visual grading score, as well as the timepoint of the PET-CT scan as (early) imaging may lead to false positive results, while a negative PET-CT finding can be employed as a rule out test for VGEI [21–23]. Future studies are needed to ascertain the definitive role of PET-CT in the diagnostic algorithm for VGEI.

The large majority of patients in our cohort were treated conservatively. Recent studies support that a conservative treatment approach may lead to similar outcomes as a surgical approach in cases without fistulation or pseudoaneurysm formation [5, 15–17], contradicting the 2020 guidelines from the European Society for Vascular Surgery on VGEI [24]. However, of note, our treatment decisions (conservative versus surgical) were based on careful patient evaluation and MDT-based decision making and outcomes can therefore not be directly compared. A conservative treatment strategy necessitates careful selection of both empirical and targeted antimicrobial therapy. At Copenhagen University Hospital, the currently recommended initial empirical treatment for VGEI is the combination of vancomycin and high-dose meropenem, which about a third of those treated empirically in our study received [25]. For comparison, in the Central Denmark Region, empirical treatment consists of ciprofloxacin in combination with standard-dose meropenem [26]. In the Netherlands, the recommended empirical treatment is a combination of piperacillin/tazobactam, vancomycin, and caspofungin [7], while in France cefotaxime and gentamicin are recommended [5]. Of note, these recommendations show heterogenicity considering the similarity of causative organisms and the known background epidemiology resistance rates across Denmark, the Netherlands and to some degree France [27]. A recent consensus document highlights the importance for guiding the choice of empirical treatment based on the graft location as well as the presence of an AEF. For aVGEI, it is recommended to use broad spectrum antibiotics to cover both Gram-positive cocci and Gram-negative bacilli [6].

In our study, the causative microorganism could be identified for the majority of patients and are in line with findings from other cohorts from Europe, with *S. aureus*, CoNS, and *Salmonella* species being the most frequently isolated microorganisms in monomicrobial infections. About a quarter of infections were polymicrobial, corresponding to other cohorts that find polymicrobial infections in 16%-35% of cases [15–18, 28]. The high morbidity and mortality of aVGEI, the risk for multi-resistant nosocomial infections, the high prevalence of *S. aureus* and anaerobic bacteria as well as propensity for biofilm formation require careful selection of empiric antibiotic regimens and early switch to a definitive and more targeted antimicrobial treatment based on microbiological results. The low prevalence of drug-resistant bacteria in Denmark confirmed by our study [29] does not necessitate empiric coverage of extended-spectrum beta-lactamase, carbapenemase-producing organisms and methicillin-resistant *S. aureus*, although larger cohorts are desirable to confirm this.

The optimal duration of antimicrobial therapy remains unclear and lifelong suppressive treatments are often considered and is highly dependent on whether surgical treatment has been performed and the type of procedure [24]. In our study, less than 40% of conservatively treated patients were selected for lifelong suppressive therapy by the MDT conference, in line with findings from France [16]. The median duration of parenteral antimicrobial therapy (30 days) was at the lower range of recommendations in local and international guidelines, which usually recommend at least four to six weeks of IV antimicrobial therapy if the graft can be removed [24, 25]. A large multi-centre study on antibiotic treatment for infectious endocarditis (IE), the POET study, showed that early peroral treatment is non-inferior compared to the standard six weeks of IV antibiotics in clearly defined circumstances including identification of the causative microorganism and stabilisation of the patient [30]. No prospective studies have so far evaluated an early switch to oral antimicrobial therapy for clinically stable VGEI patients with an identified pathogen, similar to POET criteria [30]. We speculate whether the knowledge and findings from research in IE may be transferable to the VGEI population. Indeed, we found that patient characteristics in our cohort were not different to those in the large nationwide Danish registry of patients with IE, the large National Danish Endocarditis Studies registry [31, 32]. Both cohorts were predominantly male (65.7% in aVGEI vs. 68.3% in IE) with a similar median age (73 years in aVGEI vs. 73.7 years in IE) and prevalence of major comorbidities, e.g., diabetes (25.7% in aVGEI vs. 23.7% in IE), renal disease (20% vs. 17.4%), heart failure (20% vs. 18.7%), and active malignancy (11.4% vs. 8.6%). In contrast, pulmonary disease was more frequent in aVGEI (37.1% vs. 14.3%) [32].

### Limitations

The study has some important limitations including the retrospective design, the small study population, and the fact that data was not collected at fixed time points but done as a part of routine clinical practice resulting in non-systematic follow-up. As this resulted in lack of full follow-up on some patients, this may impact the outcomes in either a positive or negative direction. Furthermore, the study exclusively included patients eligible for an MDT conference and some patients may not have been included when treated locally at other facilities or having an acutely fatal outcome and died before discussion at the MDT conference. This introduces selection bias, likely leading to underestimation of mortality and morbidity in our study that may impede generalizability. The MDT in this study consisted of vascular surgeons, infectious disease specialists, and microbiologists although a recent Delphi consensus paper suggests including nuclear medicine specialists, radiologists, and anaesthesiologists as well [12]. Applying MAGIC criteria in retrospect was also not blinded to outcomes. Although this may have introduced classification bias, any resulting bias would be expected to increase rather than decrease the proportion of patients fulfilling the criteria.

## Conclusion

In summary, this study describes a Danish cohort of patients with aVGEI characterized by advanced age and high multimorbidity, comparable to other European and US cohorts. A quarter of diagnoses did not fulfill MAGIC criteria. PET-CT provided essential diagnostic support, although its role requires further validation. At follow-up, 90-day and one-year mortality were relatively low at 8.6% and 27.3%, respectively, likely reflecting the benefits of individualized, MDT-based treatment decisions as well as survivor bias but these findings require confirmation in larger cohorts. Curative conservative management proved feasible in carefully selected patients. Due to the multimorbidity, high variability of radiological and microbiological findings, the need for surgical expertise as well as relatively low incidence of aVGEI, individualised treatment decisions, including those related to antimicrobials, should be made by an MDT conference.

## Supplementary Information

Below is the link to the electronic supplementary material.

Supplementary Material 1

## Data Availability

The data generated and analysed in this study are not publicly available due to ethical considerations but can be available from the corresponding author on reasonable request. Access may be granted in accordance with an institutional review board.

## Footnotes

1 Samples from wound included swab (*n* = 3), secretion/pus (*n* = 4), and wound tissue (*n* = 1).
