# Supplementary material for: Multidisciplinary management and outcomes of abdominal vascular graft and endograft infections: a retrospective cohort study
Source: Infection. 2026 Jul 15;54(5):2589–600. doi: 10.1007/s15010-026-02885-w (PMC13727878; doi:10.1007/s15010-026-02885-w)
Supplement: Supplementary file 1 [file 15010_2026_2885_MOESM1_ESM.docx]

**Supplementary Material**

**Supplementary Table 1:** Strengthening the Reporting of Observational Studies in Epidemiology (STROBE) checklist for cohort studies

|  | **Item No** | **Recommendation** | **Page No** |
| --- | --- | --- | --- |
| **Title and abstract** | 1 | (*a*) Indicate the study’s design with a commonly used term in the title or the abstract | 1 |
|  |  | (*b*) Provide in the abstract an informative and balanced summary of what was done and what was found | 1-2 |
| **Introduction** |  |  |  |
| Background/rationale | 2 | Explain the scientific background and rationale for the investigation being reported | 2-3 |
| Objectives | 3 | State specific objectives, including any prespecified hypotheses | 3 |
| **Methods** |  |  |  |
| Study design | 4 | Present key elements of study design early in the paper | 3 |
| Setting | 5 | Describe the setting, locations, and relevant dates, including periods of recruitment, exposure, follow-up, and data collection | 3 |
| Participants | 6 | (*a*) Give the eligibility criteria, and the sources and methods of selection of participants. Describe methods of follow-up | 3-4 |
|  |  | (*b*) For matched studies, give matching criteria and number of exposed and unexposed | - |
| Variables | 7 | Clearly define all outcomes, exposures, predictors, potential confounders, and effect modifiers. Give diagnostic criteria, if applicable | 4-5 |
| Data sources/ measurement | 8 | For each variable of interest, give sources of data and details of methods of assessment (measurement). Describe comparability of assessment methods if there is more than one group | 5 |
| Bias | 9 | Describe any efforts to address potential sources of bias | 11 |
| Study size | 10 | Explain how the study size was arrived at | 3 |
| Quantitative variables | 11 | Explain how quantitative variables were handled in the analyses. If applicable, describe which groupings were chosen and why | 5 |
| Statistical methods | 12 | (*a*) Describe all statistical methods, including those used to control for confounding | 5 |
|  |  | (*b*) Describe any methods used to examine subgroups and interactions | 5 |
|  |  | (*c*) Explain how missing data were addressed | 5 |
|  |  | (*d*) If applicable, explain how loss to follow-up was addressed | 4 |
|  |  | (*e*) Describe any sensitivity analyses | - |
| **Results** |  |  |  |
| Participants | 13 | (a) Report numbers of individuals at each stage of study—eg numbers potentially eligible, examined for eligibility, confirmed eligible, included in the study, completing follow-up, and analysed | 5 |
|  |  | (b) Give reasons for non-participation at each stage | 5 |
|  |  | (c) Consider use of a flow diagram | 6 |
| Descriptive data | 14 | (a) Give characteristics of study participants (eg demographic, clinical, social) and information on exposures and potential confounders | 6-7 |
|  |  | (b) Indicate number of participants with missing data for each variable of interest | 7 |
|  |  | (c) Summarise follow-up time (eg, average and total amount) | 8 |
| Outcome data | 15 | Report numbers of outcome events or summary measures over time | 8-9 |
| Main results | 16 | (*a*) Give unadjusted estimates and, if applicable, confounder-adjusted estimates and their precision (eg, 95% confidence interval). Make clear which confounders were adjusted for and why they were included | 8-9 |
|  |  | (*b*) Report category boundaries when continuous variables were categorized | 7-10 |
|  |  | (*c*) If relevant, consider translating estimates of relative risk into absolute risk for a meaningful time period | - |
| Other analyses | 17 | Report other analyses done—eg analyses of subgroups and interactions, and sensitivity analyses | 10-13 |
| **Discussion** |  |  |  |
| Key results | 18 | Summarise key results with reference to study objectives | 14-16 |
| Limitations | 19 | Discuss limitations of the study, taking into account sources of potential bias or imprecision. Discuss both direction and magnitude of any potential bias | 16 |
| Interpretation | 20 | Give a cautious overall interpretation of results considering objectives, limitations, multiplicity of analyses, results from similar studies, and other relevant evidence | 16 |
| Generalisability | 21 | Discuss the generalisability (external validity) of the study results | 16 |
| **Other information** |  |  |  |
| Funding | 22 | Give the source of funding and the role of the funders for the present study and, if applicable, for the original study on which the present article is based | 17 |

**Supplementary Table 2a.** Microbiology among ‘MAGIC positive aVGEI’ patients

|  | **Microbiology from culture** | **Localization of specimen** | **Empirical treatment** | **Time from diagnosis to targeted treatment** | **Targeted treatment (IV)** | **Period of IV treat-ment** | **Peroral antibiotics** | **Total duration of antibiotics**  **(suppressive vs. curative)** | **Outcome^12^**  **(follow-up period from aVGEI diagnosis)** | |
| --- | --- | --- | --- | --- | --- | --- | --- | --- | --- | --- |
| **Early infection** | |  |  |  |  |  |  |  | |  |
| I | *Staphylococcus aureus* | Blood culture | No | 0 days | Cefuroxime and p.o. rifampin | 42 days | Dicloxacillin | Lifelong  (suppressive) | | Insufficient follow-up  (267 days) |
| II | *Candida tropicalis*, *Lactobacillus fermentum*, *Escherichia coli,* and non-hemolytic *streptococcus* | Graft material* | Meropenem, metronidazole, and fluconazole | 16 days | Ampicillin, ciprofloxacin, and fluconazole | 40 days | Amoxicillin, ciprofloxacin, and fluconazole | Lifelong (suppressive) | | Remission |
| III | *S. aureus* | Blood culture before insertion of graft | Meropenem and vancomycin | 0 days | Meropenem | 19 days | Sulfametoxazole and trimetoprim (SMX-TMP) | 28 days  (curative) | | Cure |
| IV | *Listeria monocytogenes* | Blood culture and aneurysm tissue sample | No | 0 days | Ampicillin, gentamicin, and p.o. rifampin | 15 days | Rifampin (31 days) and amoxicillin (368 days) | 383 days  (suppressive) | | Remission |
| V | *S. aureus* | Blood culture | No | 0 days | Cefuroxime and p.o. rifampin | 31 days | Rifampin and moxifloxacin | 350 days  (curative) | | Remission |
| VI | Negative culture  (PCR: *Streptococcus mitis* and *Anaerococcus* species) | Aneurysm tissue sample | Ampicillin and gentamicin | 0 days | Meropenem and vancomycin | 35 days | Metronidazole and amoxicillin | 62 days  (curative) | | Insufficient follow-up  (283 days) |
| VII | *S. aureus*, *Acinetobacter baumanii* complex, *Klebsiella oxytoca* complex and hemolytic *streptococcus* group B | Wound swab | Cefuroxime | 2 days | Meropenem and rifampin | 24 days | Ciprofloxacin, metronidazole, and amoxicillin/ clavulanic acid | 42 days  (curative) | | Cure |
| VIII | *Streptococcus pneumoniae* | Blood culture | No | 0 days | Ceftriaxone | 62 days | Moxifloxacin | 182 days  (curative) | | Cure |
| IX | Unknown |  | Meropenem, metronidazole, and ciprofloxacin |  | No | 61 days | Ertapenem, ciprofloxacin, and amoxicillin/clavulanate | 305 days  (curative) | | Insufficient follow-up  (435 days) |
| X | Unknown  (PCR: Negative) | Aneurysm tissue | Meropenem and vancomycin |  | No | 11 days | Moxifloxacin | 16 days  (curative) | | Insufficient follow-up  (174 days) |
| XI | *Staphylococcus capitis* | Aneurysm tissue | Vancomycin and rifampin | 6 days | Cloxacillin and p.o. rifampin | 16 days | Dicloxacillin and rifampin | 42 days  (curative) | | Cure |
|  | *S. epidermidis* | Blood culture |  |  |  |  |  |  |  |  |
| XII | *E. coli* | Secretion from wound | Cefuroxime | 3 days | Ceftriaxone | 8 days | Ciprofloxacin and metronidazole, fluconazole for 7 days | 34 days  (curative) | | Insufficient follow-up  (356 days) |
| XIII | Unknown  (PCR: Negative) |  | Meropenem and vancomycin |  | No | 33 days | Moxifloxacin and linezolid | 50 days  (curative) | | Insufficient follow-up  (194 days) |
| **Late infection** | |  |  |  |  |  |  |  | |  |
| XIV | *Cutibacterium acnes* | Aneurysm tissue sample and aspirate | Meropenem and vancomycin | 0 days | Meropenem and rifampin | 29 days | Moxifloxacin (32 days) followed by amoxicillin (65 days) and lastly doxycykline | Lifelong (suppressive) | | Remission |
| XV | *E. coli*  (PCR: *Parvimonas* species and *E. coli* from pus) | Blood culture | No | 0 days | Pipercillinam/ tazobactam, metronidazole, fluconazole (7 days), and ciprofloxacin | 21 days | Ciprofloxacin and metronidazole | Lifelong (suppressive) | | Death |
| XVI | Unknown |  | Meropenem |  |  | 3 days |  | 3 days  (curative) | | Death |
| XVII | *Enterococcus faecium*  (VLRE)  (PCR: *Lactobacillus*) | Blood culture and wound swab | No | 0 days | Meropenem, linezolid, anidulafungin, and ciprofloxacin | 316 days | SMX-TMP, fluconazole, and oritavancin^a^ | Lifelong  (curative) | | Insufficient follow-up  (285 days) |
|  | *E. coli*, *Candida Glabrata* and *Klebsiella* | Swab from aneurysm |  |  |  |  |  |  |  |  |
| XVIII | Negative culture  (PCR: *Streptococcus dysgalactiae*) | Stent graft material | Cefuroxime | 4 days | p.o moxifloxacin and amoxicillin | 4 days | Moxifloxacin and amoxicillin | 28 days (curative) | | Insufficient follow-up  (297 days) |
| XIX | *S. aureus* | Blood culture and secretion from wound | Vancomycin and meropenem | 0 days | Benzylpenicillin and p.o rifampin | 50 days | Amoxicillin/ clavulanic acid (168 days) followed by amoxicillin | Lifelong  (suppressive) | | Remission |
| XX | *Corynebacterium jeikeium* | Graft material | No | 0 days | Meropenem and linezolid | 4 days | Moxifloxacin and linezolid | 46 days  (curative) | | Cure |
|  | *E. facaelis* | Wound tissue |  |  |  |  |  |  |  |  |
| XXI | *S. epidermidis* and *Peptoniphilus harei*  (PCR: CoNS and *P. harei*) | Aspirate | No | 0 days | Vancomycin, metronidazoland piperacillin/tazobactam | 20 days | Metronidazol (349 days) and dicloxacillin (474 days) | 502 days  (suppressive) | | Remission |
| XXII | *E. coli* | Blood culture* | No | 0 days | Meropenem | 5 days |  | 5 days  (curative) | | Death |
| XXIII | *Staphylococcus lugdunensis* | Pus | Meropenem and ciprofloxacin | 0 days | Benzylpenicillin (18 days) and p.o. rifampin, later cefuroxime (6 days) | 31 days | Amoxicillin (12 days) and rifampin followed by ciprofloxacin (16 days) | 59 days  (curative) | | Insufficient follow-up  (203 days) |
| XXIV | Negative culture  (PCR: *S. epidermidis* og *C. acnes*) | Graft tissue | Meropenem and linezolid |  |  | 50 days | Ertapenem^b^ and linezolid | 52 days  (curative) | | Insufficient follow-up  (375 days) |
| XXV | *Salmonella* species | Graft tissue and blood culture* | Meropenem and ciprofloxacin | 4 days | Ampicillin and ciprofloxacin | 15 days |  | 15 days  (curative) | | Death |
| XXVI | Negative culture  (PCR: CoNS) | Wound swab and fluid^c^ | No | 11 days | p.o. dicloxacillin and p.o. rifampin (6 days) | 0 days | Dicloxacillin | 279 days (suppressive) | | Death |
| XXVII | *Campylobacter fetus* | Blood culture | Meropenem and vancomycin | 2 days | Meropenem and ciprofloxacin | 51 days | Ciprofloxacin | Lifelong  (suppressive) | | Remission |

**Fistula present.
Abbreviations: PCR: Polymerase Chain Reaction (16S ribosomal RNA analysis was used). VLRE: Vancomycin and Linezolid resistant enterococcus. CPO: Carbapenemase-Producing Organism. CoNS: Coagulase negative Staphylococci*

^a^ Treatment was given as IV treatment once a week after discharge

^b^ Treatment was given as IV treatment at home

^c^ From skin fistula formation

**Supplementary Table 2b.** Microbiology among ‘clinical aVGEI’ patients by MDT consensus

|  | **Microbiology from culture** | **Localization of specimen** | **Empirical treatment** | **Time from diagnosis to definite treatment** | **Definite treatment** | **Time from start of treatment to peroral treatment** | **Peroral antibiotics** | **Total duration of antibiotics**  **(suppressive vs. curative)** | **Outcome^12^**  **(follow-up period from aVGEI diagnosis)** |
| --- | --- | --- | --- | --- | --- | --- | --- | --- | --- |
| **Early infection^a^** | |  |  |  |  |  |  |  |  |
| I | *S. epidermidis* | Hematoma in groin | Meropenem, metronidazole, and fluconazole | 15 days | Linezolid | 44 days | Moxifloxacin | 55 days  (curative) | Cure |
| II | Unknown |  | Meropenem and vancomycin | 8 days | Cefuroxime and linezolid | 40 days | None | 40 days  (curative) | Insufficient follow-up  (156 days) |
| **Late infection^a^** | |  |  |  |  |  |  |  |  |
| III | *S.aureus* | Blood culture | No | 2 days | Benzylpenicillin and p.o. rifampin | 20 days | Amoxicillin (23 days) and rifampin | 42 days  (curative) | Treatment failure |
| IV | *Salmonella* species | Blood culture | No | 0 days | Ceftriaxone | 44 days | Azithromycin (113 days) followed by SMX-TMP (88 days) | 245 days  (curative) | Cure |
| V | Unknown |  | Cefuroxime |  | No | 27 days | Moxifloxacin (111 days) followed by amoxicillin/ clavulanic acid | Lifelong  (suppressive) | Insufficient follow-up  (217 days) |
| VI | *S. mitis* | Blood culture | No | 2 days | Benzylpenicillin and rifampin | 23 days | Amoxicillin and rifampin | Lifelong  (suppressive) | Remission |
| VII | *S. epidermidis* | Blood culture | Meropenem and vancomycin | 34 days | Meropenem, vancomycin, and p.o. linezolid^b^ | 53 days | Metronidazol, linezolid, and ceftazidime/ avibactam^c^ | 53 days  (suppressive) | Death |
| VIII | Unknown |  | Meropenem and vancomycin |  | No | 16 days | Moxifloxacin and linezolid (42 days) | Lifelong  (suppressive) | Insufficient follow-up  (156 days) |

^a^ As MAGIC criteria were not fulfilled; date of diagnosis was sat at date of suspicion of aVGEI

^b^ Patient was given six days of peroral treatment with moxifloxacin, doxycycline and fluconazol before returning to this regime

^c^ Treatment was given as IV treatment at home

**Supplementary Table 3.** Details on surgical treatment of aVGEI patients

| **Patient** | **Age** | **CCI** | **Index surgery** | **Localiazation of graft** | **Type of surgery** | **Time from index surgery to surgical treatment** | **Time from diagnosis to surgical treatment** | **Outcome** |
| --- | --- | --- | --- | --- | --- | --- | --- | --- |
| XV | 67 | 3 | Endovascular repair | Abdominal aorta | Endovascular repair | 469 days | 3 days | Death |
| XVII | 74 | 3 | Open surgical repair with Dacron graft | Aortobiiliacal | Total graft removal with in situ reconstruction using bovine pericardium graft | 461 days | 30 days | Insufficient follow-up |
| XVIII | 49 | 0 | Endovascular repair | A. iliaca externa | Partial graft removal with in situ reconstruction using autologous vein graft | 2,965 days | 0 days | Insufficient follow-up |
| XXIII | 62 | 3 | Endovascular repair | A. iliaca externa | Partial graft removal with in situ reconstruction using autologous vein graft | 254 days | 15 days | Insufficient follow-up |
| XXIV | 70 | 5 | Open surgical repair with Dacron graft | Aorto-bifemoral | Total graft removal with in situ reconstruction using bovine pericardium graft | 1,684 days | 9 days | Insufficient follow-up |

*Abbreviations: CCI: Charlson Comorbidity Index. EAB: Extra-anatomical bypass*

**Supplementary Figure 1.** Kaplan-Meier plot illustrating overall one-year survival among all aVGEI patients.

**Supplementary Figure 2.** Kaplan-Meier plot illustrating overall one-year survival among ‘MAGIC positive aVGEI’ patients without an aortoenteric fistula.
